# An Unusual Orthopaedic Disease: Sarcoidosis—A Case Report

**DOI:** 10.1055/s-0037-1601879

**Published:** 2017-04-18

**Authors:** Bahattin Kemah, Burak Özturan, Bilge Bilgic, Korhan Özkan, Fuat Akpınar, Bulent Kılıc

**Affiliations:** 1 Department of Orthopaedics and Traumatology, Agri State Hospital, Agri, Turkey; 2 Department of Orthopaedics and Traumatology, Istanbul Medeniyet University Göztepe Training and Research Hospital, Istanbul, Turkey; 3 Department of Pathology, Istanbul Medicine Faculty, Istanbul University, Istanbul, Turkey; 4 Department of Orthopaedics and Traumatology, Istanbul Gelisim University, Istanbul, Turkey

**Keywords:** sarcoidosis, bone, granulomatous disease

## Abstract

Sarcoidosis is an idiopathic, noncaseating granulomatous disorder with wide systemic involvement. It is encountered widely around the world and it affects both sexes, all the races in all age groups. Lungs, eyes, and skin are the organs most commonly affected. Constitutional features such as weight loss, fatigue, and myalgia are the most common symptoms. Bone involvement, which is very rare, was reported as present in 3 to 13% of effected cases, and it is most commonly seen in hands and feet, compared with long bone involvement, which is extremely rare. We hereby present a case with a diagnosis of sarcoidosis and multiple bone involvement emphasizing the importance of differential diagnosis.

Sarcoidosis is an autoimmune disorder of unknown etiology with noncaseating granulomatous inflammation and multiple organ and system involvement. It is more common in individuals younger than the age of 50 years with a slight predominance in females. 1 While involvements of lungs, respiratory system, and skin are more common, multiple involvement of lymph nodes, salivary glands, eyes, musculoskeletal system may also be seen. The pathophysiology of the disease is not exactly discovered and the diagnosis is made after pathologic investigations. Bone involvement of sarcoidosis is first described by Kreibich after detecting multiple radiolucent areas in distal phalanges of second finger in four patients in 1904. 2 Bone involvement of sarcoidosis is generally asymptomatic and its frequency ranges from 3 to 13%. 1 3 Bone involvement may be lytic, sclerotic, or mixed type. 4 Radiologic findings include the appearance of well-demarcated cysts without any periosteal reaction and no peripheral sclerosis. 1 The disease involves mostly phalanges in the skeletal system, forming dactylitis. The involvement may also be present in maxilla, skull, facial bones, vertebrae, ribs, and pelvic bones 5 ; however, infrequent involvement of other bones, especially long ones might be encountered as in the case presented here. The diagnosis is made with the help of findings related to systemic involvement, solitary bone lesions, and after the bone biopsy.

In this report, we aimed to present a case with sarcoidosis and bone involvement and emphasize on the importance of differential diagnosis because sarcoidosis can mimic any granulomatous disease, primary or metastatic cancers to the bone.

## Case Presentation

**Fig. 1:**
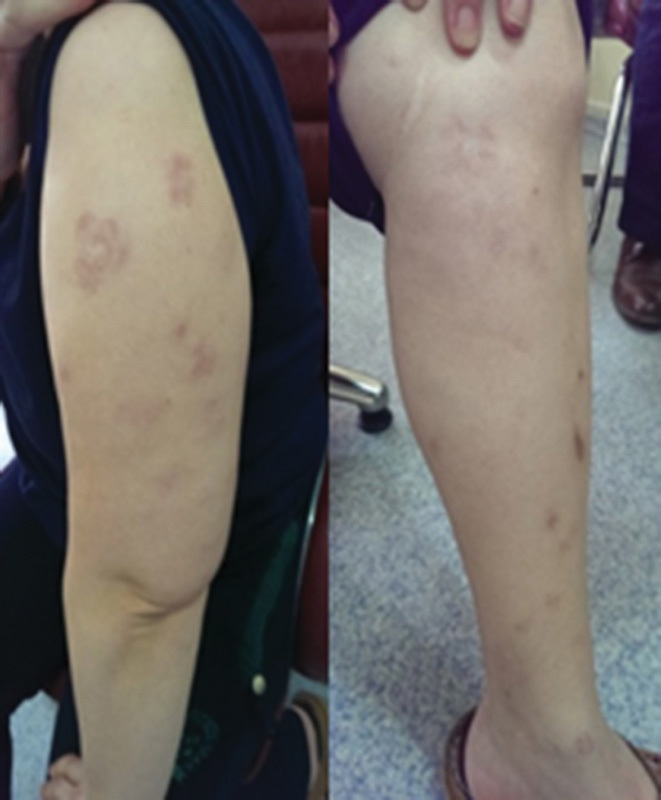
Erythematous nodules on arm and leg.

**Fig. 2:**
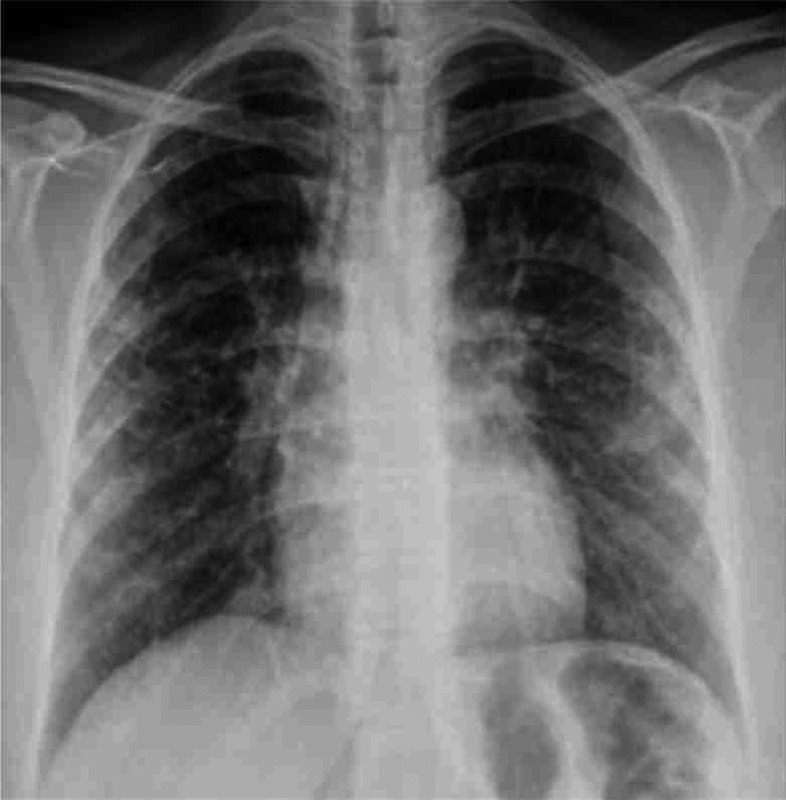
Lung X-ray (after treatment).

**Fig. 3:**
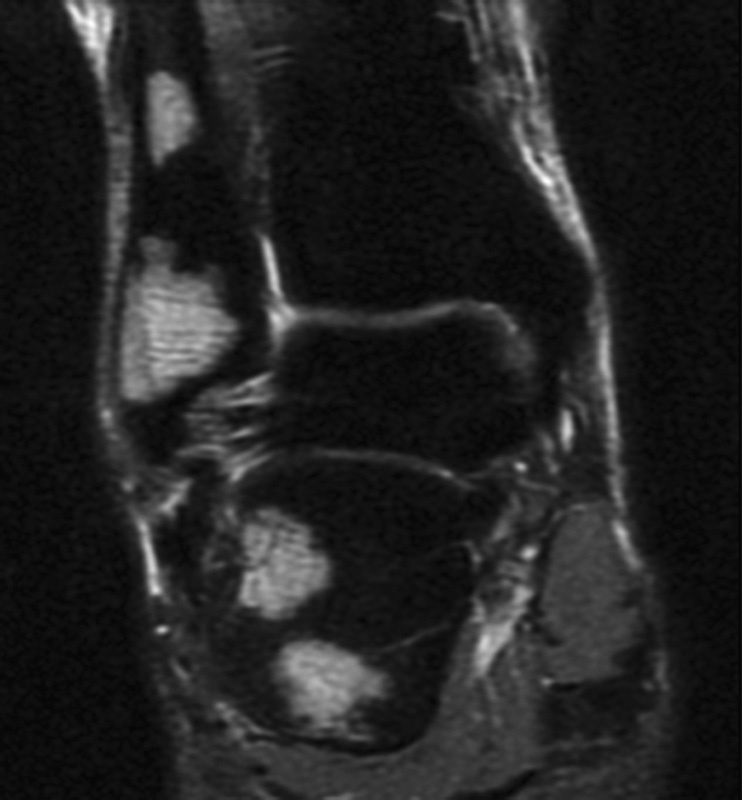
Hyperintense lesions in fibula and calcaneus (T2-weighted magnetic resonance imaging section).

A 44-year-old female patient was admitted to our outpatient clinic due to complaint of right ankle pain. The patient, who described the pain as continuous during the day unrelated to physical activity, informed us about the past medical history of sarcoidosis. She had applied to a health care facility due to pale and nodular lesions present on trunk, upper and lower extremities 8 years ago ( Fig. 1 ). We found out that the biopsy results of those lesions showed granulomatous dermatitis, and that no definitive diagnosis had been made. Her radiologic work-up (X-ray, positron emission tomography–computed tomography [PET-CT]) and lung biopsy after complaints of shortness of breath showed sarcoidosis and she was started on appropriate therapy 5 years ago ( Fig. 2 ). The patient was treated with systemic corticosteroids, methotrexate, and hydroxychloroquine sulfate because of lung sarcoidosis. 6 Also, the patient was treated with roaccutane, topical methylprednisolone for skin lesion. On her application to our clinic, she was found to have lytic lesions with sclerotic points around the fibula in her ankle X-ray and further investigations were made with the help of magnetic resonance imaging (MRI). MRI showed hypointensity and hyperintensity lesions in T1- and T2-weighted sections, respectively ( Figs. 3 4 5 6 ). In PET-CT evaluation, she was found to have multiple masses with hypermetabolic activity and some lytic appearance in proximal phalange of fourth finger of left hand, right patella, distal part of both tibia, distal part of right fibula, left talus, bilateral calcaneal bones, and cuneiform bones of both feet, as well as fourth metatarsal bone of right foot, first and fourth phalanges of left foot, and first and fifth phalanges of right foot. MRI showed no soft tissue components related to these lesions. An open biopsy was performed to the right ankle on distal end of fibula and specimens of cortical and medullary bones were obtained. Pathological examination of tissue specimens showed noncaseating granulomatous inflammation compatible with sarcoidosis. After biopsy, the patient treatment continued with systemic corticosteroid and methotrexate.

**Fig. 4:**
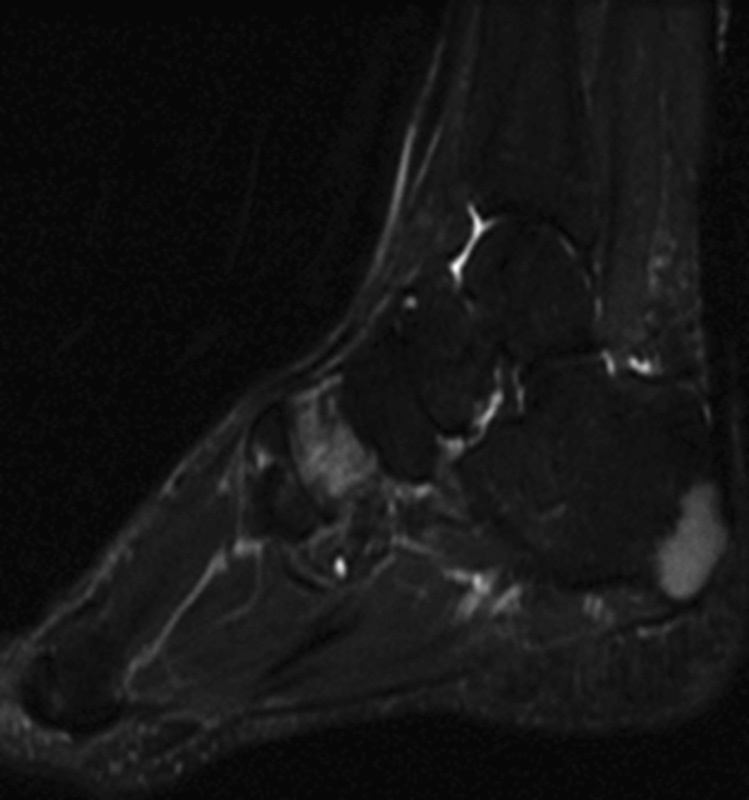
Hyperintense lesions in calcaneus and cuneiform (T2-weighted magnetic resonance imaging section).

**Fig. 5:**
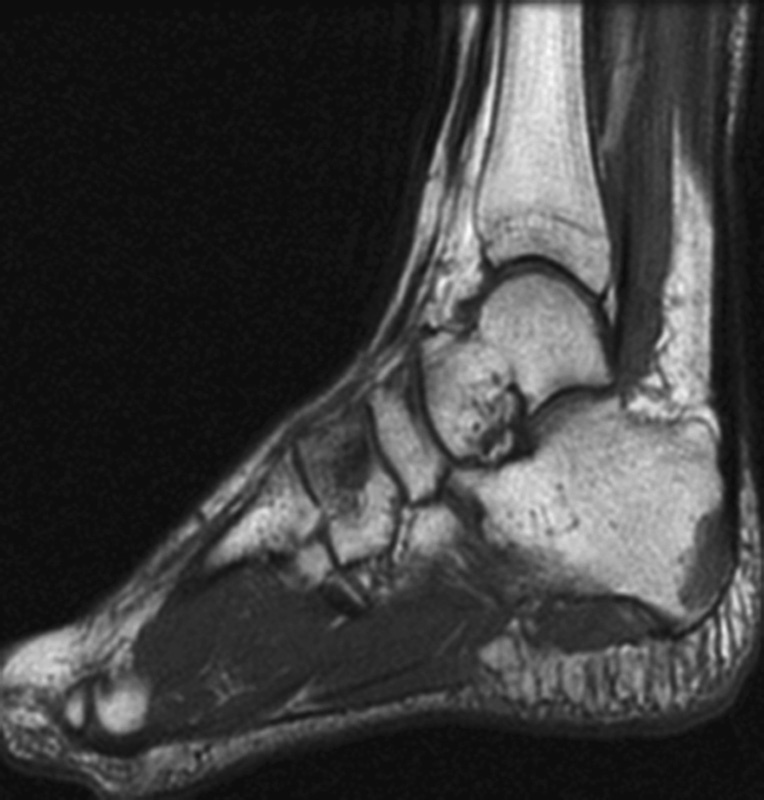
Hypointense lesions in calcaneus and cuneiform (T1-weighted magnetic resonance imaging section).

**Fig. 6:**
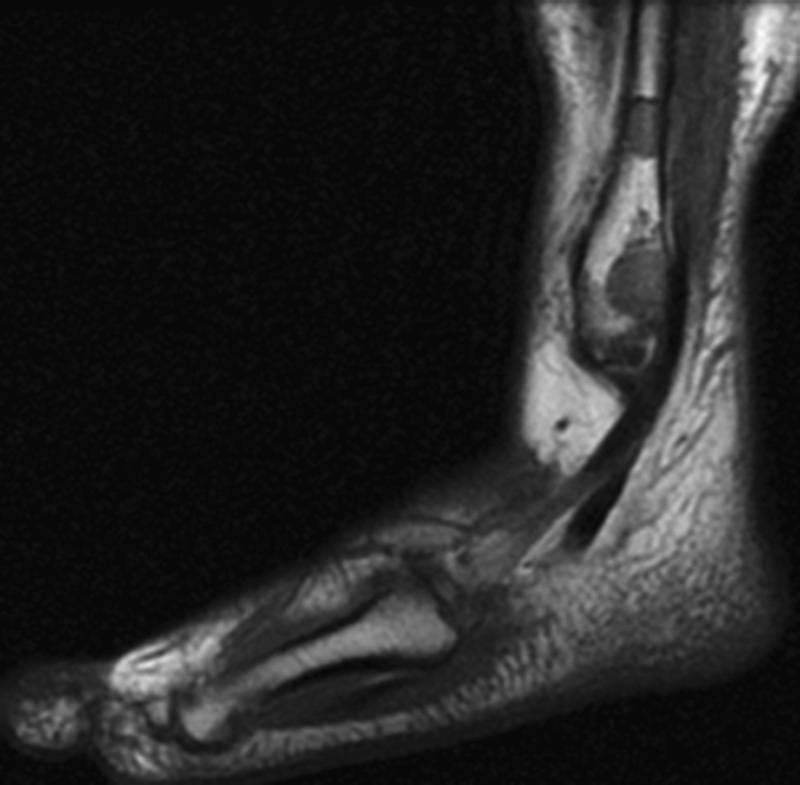
Hypointense lesions in fibula (T1-weighted magnetic resonance imaging section).

## Discussion and Conclusion

Sarcoidosis is a disease characterized by multisystemic involvement of noncaseating granulomas of unknown etiology. It is commonly encountered with lung, skin, and lymph node involvements. Total 80% of cases diagnosed with sarcoidosis are females. 7 It is more common in women and young adults younger than the age of 40 years and in African Americans and North Europeans. 8 Uveitis is the most predominant lesion in African Americans, while erythema nodosum is the most common manifestation in North Europeans. Heart and eye involvement is common in Japanese descendants and cardiac involvement is the most common cause of death. In other populations, the most common cause of death is the respiratory insufficiency due to pulmonary fibrosis. Overall mortality is between 1 and 5%. 9

An acute form of sarcoidosis is a course defined with arthritis, erythema nodosum, and bilateral hilar lymphadenopathy, called Löfgren's syndrome. 10 Locomotor system findings are mostly subclinical and they do not lead to a diagnosis by themselves; however, they might cause pain as in our case. 8 Another skeletal system findings are avascular necrosis and osteoporosis secondary to glucocorticoid use for treatment of disease. 11 12

Although the etiology of sarcoidosis is not known definitely, genetic (human leukocyte antigens) and acquired factors (air-borne antigens, viruses, fungi, and mycobacteria) play an important role. 8

Sarcoidosis generally involves skull, vertebrae, nasal bones, as well as bones in hands and feet. Lytic and sclerotic lesions have been observed in cases of vertebral sarcoidosis. Thoracic spine is the most frequently affected site in vertebral sarcoidosis. 11 Its course is asymptomatic and rarely pain may be the only manifestation. It might also involve the joints. 11 Long bone involvement is not very common. However, in our case, multiple involvement regions were present in addition to the bones of hands and feet. Sarcoidosis causes dactylitis when hand bones are involved. It usually affects second and third fingers of hands and first finger of the feet. We detected bone involvement in fourth finger of left hand in our case. Similar to the literature, involvement of first toes of bilateral feet were present. Differently, we did not detect any involvement of vertebrae and skull.

Radiologically, it may manifest as cystic or osteolytic lesions or as cortical defects and reticularizations of cortical bones as well as sclerotic and destructive lesions or similar to periostitis. 1 11 Bone involvement in sarcoidosis may even cause destructive lesions resulting in pathological fractures. 8 In MRI, T1 sequences show decreased nonspecific signal intensities, while T2 sequences show increased nonspecific signal intensities. 11 The sensitivity of MRI may be the preferred modality for the diagnosis of osseous sarcoidosis, especially when sacroiliitis is in the differential diagnosis. 6 CT images show bone destruction and sclerosis.

Other granulomatous diseases of the bone, such as Langerhans cell histiocytosis, tuberculosis with bone involvement, fungal infections, viral infections such as Epstein–Barr virus and cytomegalovirus, and Ewing sarcoma, as well as primary and secondary metastatic bone tumors must be included in differential diagnosis. 13

**Fig. 7:**
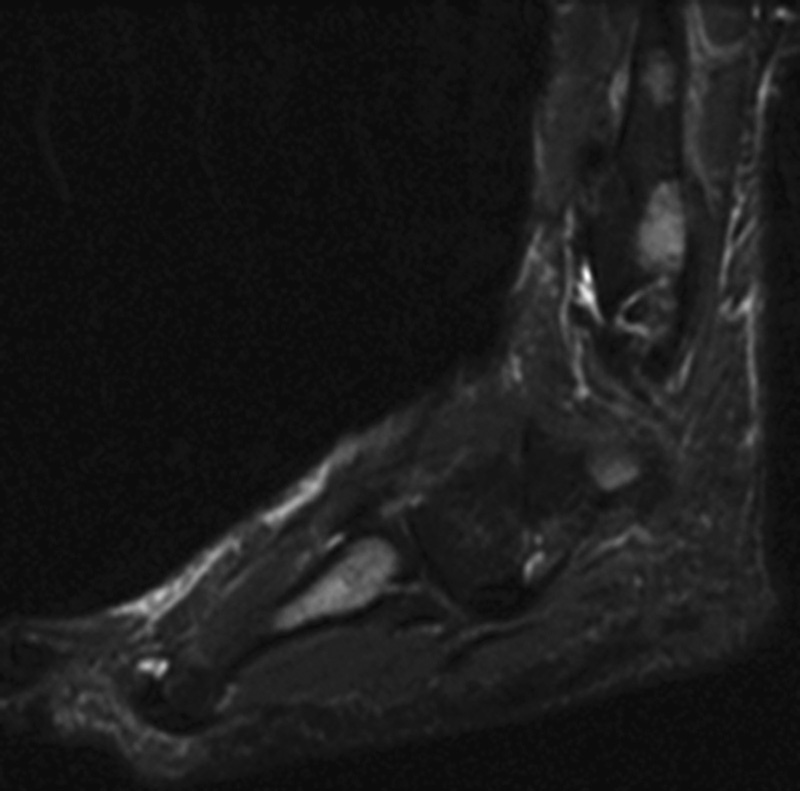
Hyperintense lesions in fibula and fourth metatarsal bone (T2-weighted magnetic resonance imaging section).

**Fig. 8:**
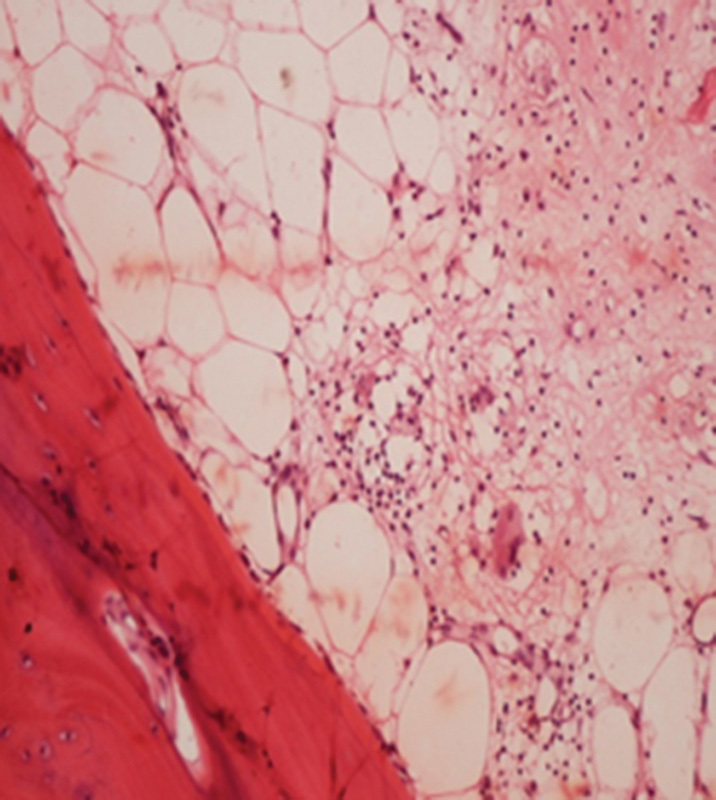
Nonnecrotizing histiocytic granulomatous lesion (×200 hematoxylin and eosin stain).

In bone biopsies, granulomas in medullary cavity and destruction in surrounding bone tissue are characteristic. 14 Nonnecrotizing histiocytic granulomas are the elementary lesion ( Figs. 7 and 8 ).

There are different drug alternatives in the treatment of sarcoidosis. There are different drug alternatives depending on the site of involvement in the body (corticosteroids, chloroquine, methotrexate, azathioprine, leflunomide, cyclosporine, pentylphenyl, minocycline, cyclophosphamide, anti-TNF, and rituximab).

Consequently, the definitive diagnosis should be made with biopsy followed by pathologic investigation. The importance of other systemic examinations and anamnesis should be emphasized to suspect of sarcoidosis. Certainly, diagnosis of sarcoidosis should be consistent systemic finding with biopsy.
